# Hip Dislocation Following Dynamic Hip Screw Fixation: A Rare Complication

**DOI:** 10.7759/cureus.102973

**Published:** 2026-02-04

**Authors:** Safa Yousif, Bharathkumar Balasubramanian, Anastasios Nikolaides

**Affiliations:** 1 Trauma and Orthopaedics, Royal Free Hospital, London, GBR; 2 Trauma and Orthopaedics, East and North Hertfordshire NHS Trust, Stevenage, GBR; 3 Trauma and Orthopaedics, University Hospitals of Birmingham NHS Foundation Trust, Birmingham, GBR

**Keywords:** dynamic hip screw, excision arthroplasty, hip dislocation, infection, intertrochanteric fracture

## Abstract

Hip dislocation is a rare complication following fixation of an intertrochanteric fracture of the proximal femur, and only a few cases of this condition have been documented in the literature. Atraumatic dislocation and deep infection are the most commonly reported. We report a case of hip dislocation presenting with pain and reduced mobility six months after dynamic hip screw (DHS) fixation. Latent deep infection was diagnosed, and organisms were isolated through intraoperative sampling. The case was managed with debridement, Girdlestone excision arthroplasty, and targeted antibiotics. Latent deep infection was identified as the cause of hip dislocation following DHS fixation in this case, with confirmation obtained via deep tissue sampling. Although spontaneous atraumatic dislocation after DHS fixation has been reported, underlying infection should be carefully ruled out.

## Introduction

Intertrochanteric fractures of the proximal femur are common in the elderly population following low-energy falls. They can also occur in younger patients after high-energy trauma. Treatment is operative, using either dynamic hip screw (DHS) fixation or cephalomedullary nail fixation, depending on the fracture pattern [1]. DHS remains a widely used and well-established method for stable extracapsular hip fractures, providing controlled fracture impaction and reliable biomechanical stability. Its relative technical simplicity, cost-effectiveness, and predictable outcomes have contributed to its continued global use.

Specific complications following DHS fixation include implant failure, screw cutout, nonunion, and malunion. Hip dislocation following DHS fixation is exceptionally rare, with only a limited number of cases reported in the literature. The mechanism underlying such dislocations remains poorly understood. Infection has been identified as an important etiological factor in previous reports [2,3]. We report a case of hip dislocation presenting six months after DHS fixation, in which deep infection was diagnosed through intraoperative sampling. This report highlights an extremely rare but clinically significant complication of a routinely performed procedure, one that many orthopaedic surgeons may never encounter in their careers.

## Case presentation

**Figure 1:**
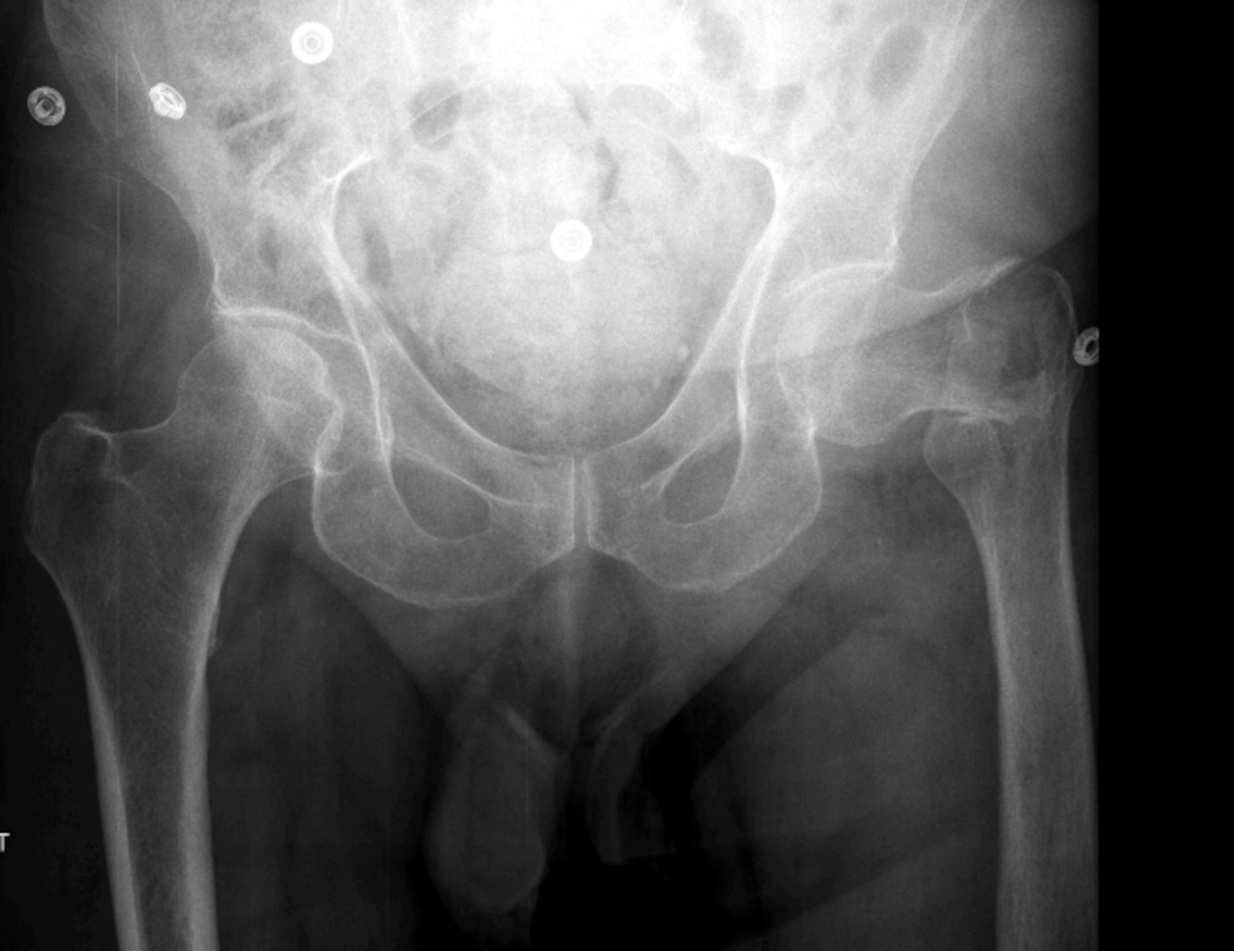
Pelvic X-ray anteroposterior view The image shows the left extracapsular intertrochanteric neck of femur fracture

**Figure 2:**
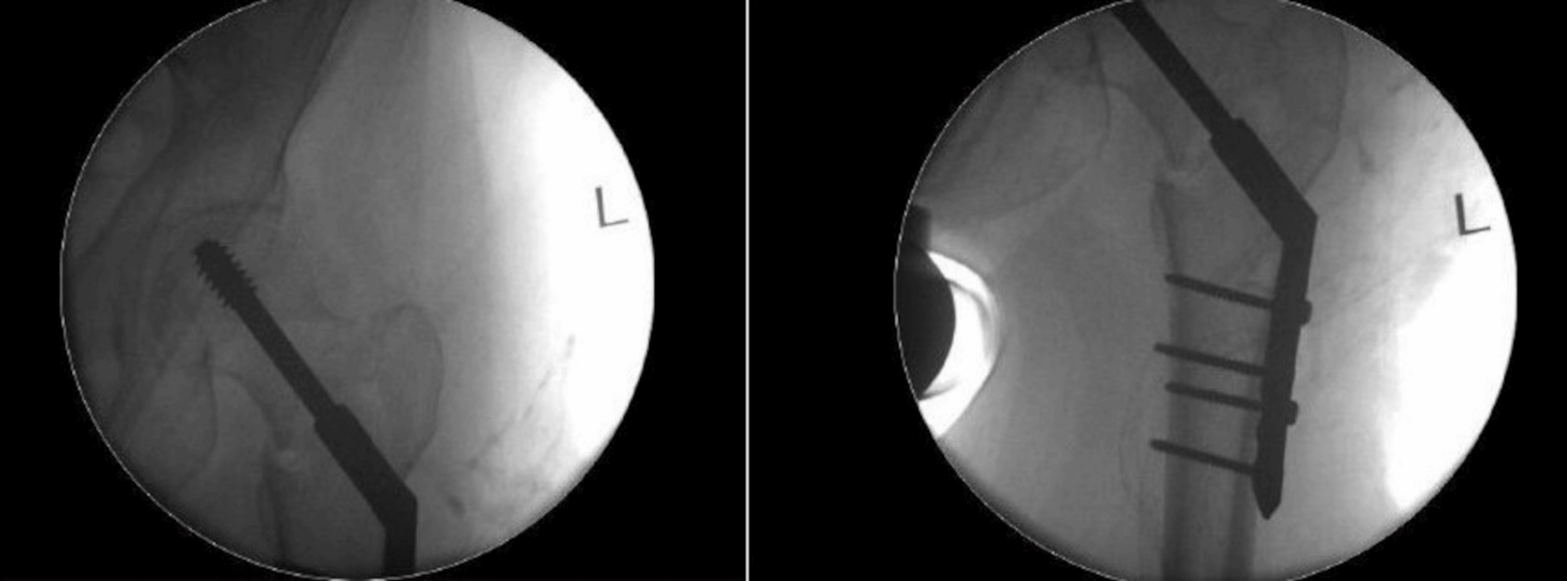
Intraoperative anteroposterior views of the left hip DHS fixation The images show adequate reduction and fixation. The view on the left centered on the hip joint, while the view on the right centered on the proximal femur DHS: dynamic hip screw; L: left

**Figure 3:**
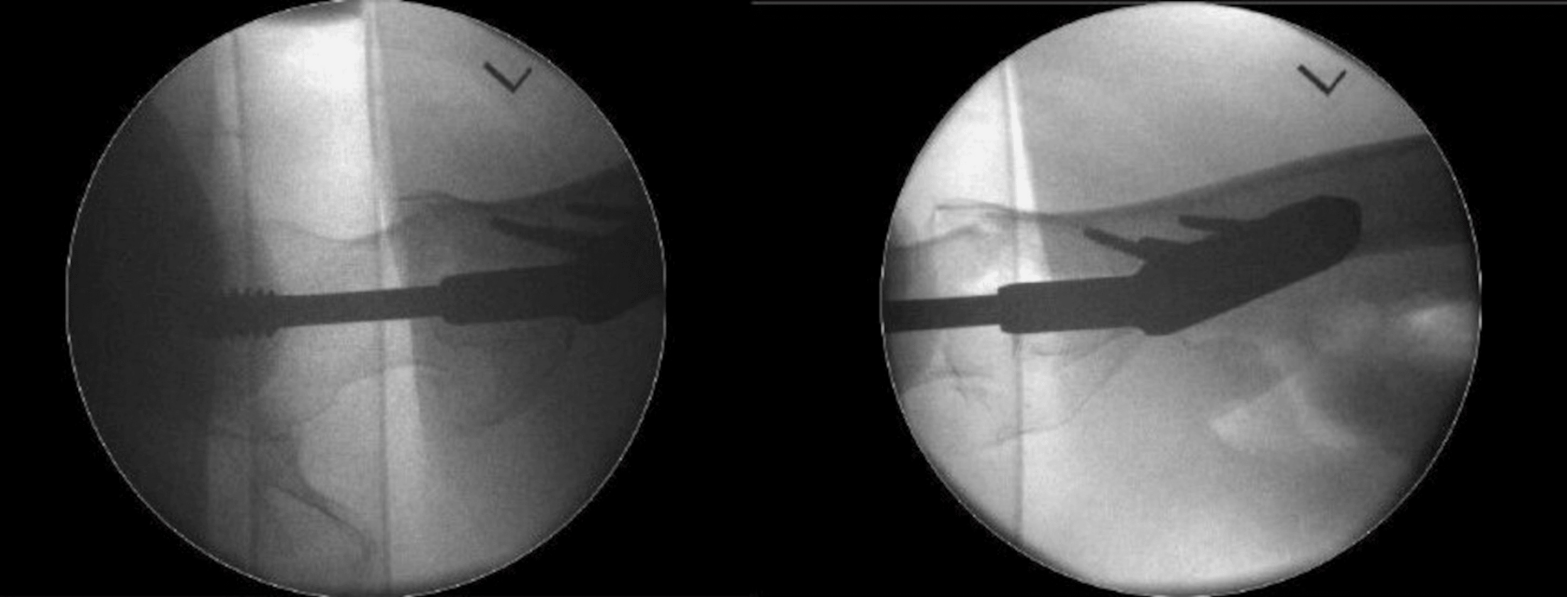
Intraoperative lateral views of the left hip DHS fixation The images show adequate reduction and fixation. The view on the left centered on the hip joint, while the view on the right centered on the proximal femur DHS: dynamic hip screw; L: left

A 57-year-old patient presented with atraumatic hip pain. The caregiver had noted a reduction in the patient’s mobility. The patient had undergone DHS fixation for a left intertrochanteric fracture six months prior (Figure 1). Intraoperative imaging at the time of fixation showed satisfactory reduction and fixation (Figures 2, 3). There were no postoperative complications. The patient’s medical history included autism and a learning disability. Indoors, the patient required single-person assistance for mobility, while outdoor mobility was dependent on a wheelchair.

**Figure 4:**
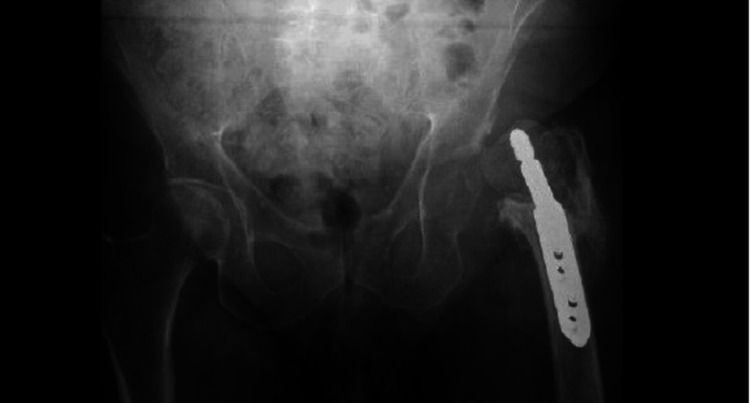
Pelvic X-ray anteroposterior view The image shows the dislocated left hip

On admission, the patient was afebrile. The left lower limb was shortened and externally rotated. Examination of the left hip revealed a healed surgical scar with no erythema or increased warmth. Blood investigations demonstrated a C-reactive protein (CRP) level of 66 mg/L (reference range: 0-5) and a white blood cell count of 5.7 × 10^9^/L (reference range 3.26-11.2). A pelvic radiograph showed dislocation of the left hip (Figure 4). The Patient had a normal chest X-ray, negative urine culture, and blood culture.

**Figure 5:**
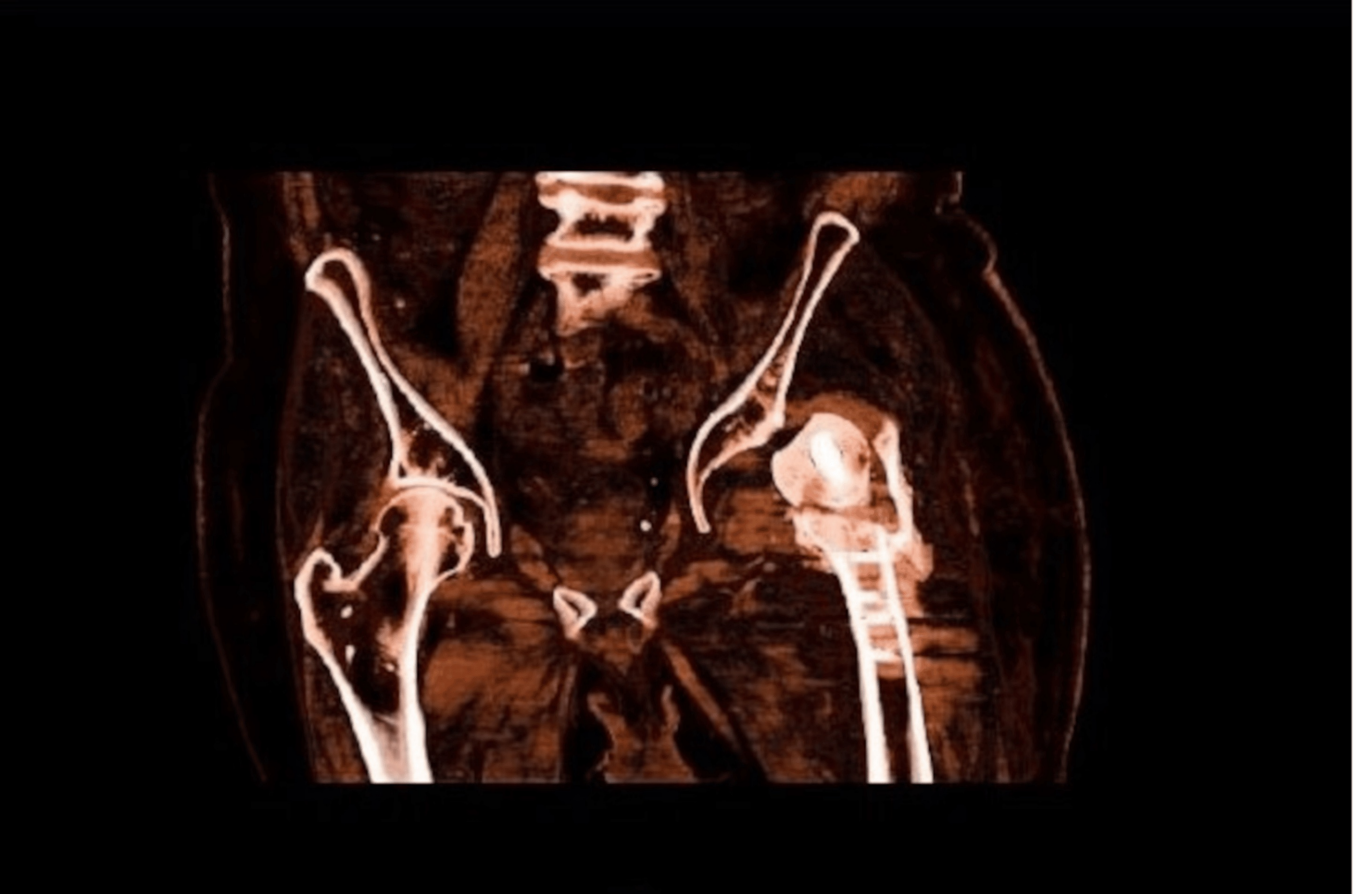
CT pelvic coronal view scan The image shows the left non-united pertrochanteic fracture, dislocated left hip, left acetabulum wall, and femoral head fracture CT: computed tomography

A CT scan of the pelvis revealed a dislocated left hip with a non-united per-trochanteric fracture, an impaction fracture of the femoral head and the superior acetabular wall, and no evidence of metalwork failure or peri-prosthetic lucency (Figure 5). A hip aspiration was attempted under imaging guidance, but it resulted in a dry tap.

The case was discussed at the prosthetic joint infection (PJI) multidisciplinary team (MDT) meeting. Management options considered included dual-mobility hemiarthroplasty or Girdlestone excision arthroplasty as a definitive procedure, depending on intraoperative findings. The patient was considered high risk for complications due to their learning disability, limited mobility, and increased muscle tone. The management plan was discussed with the patient’s relatives, and excision arthroplasty was performed.

**Figure 6:**
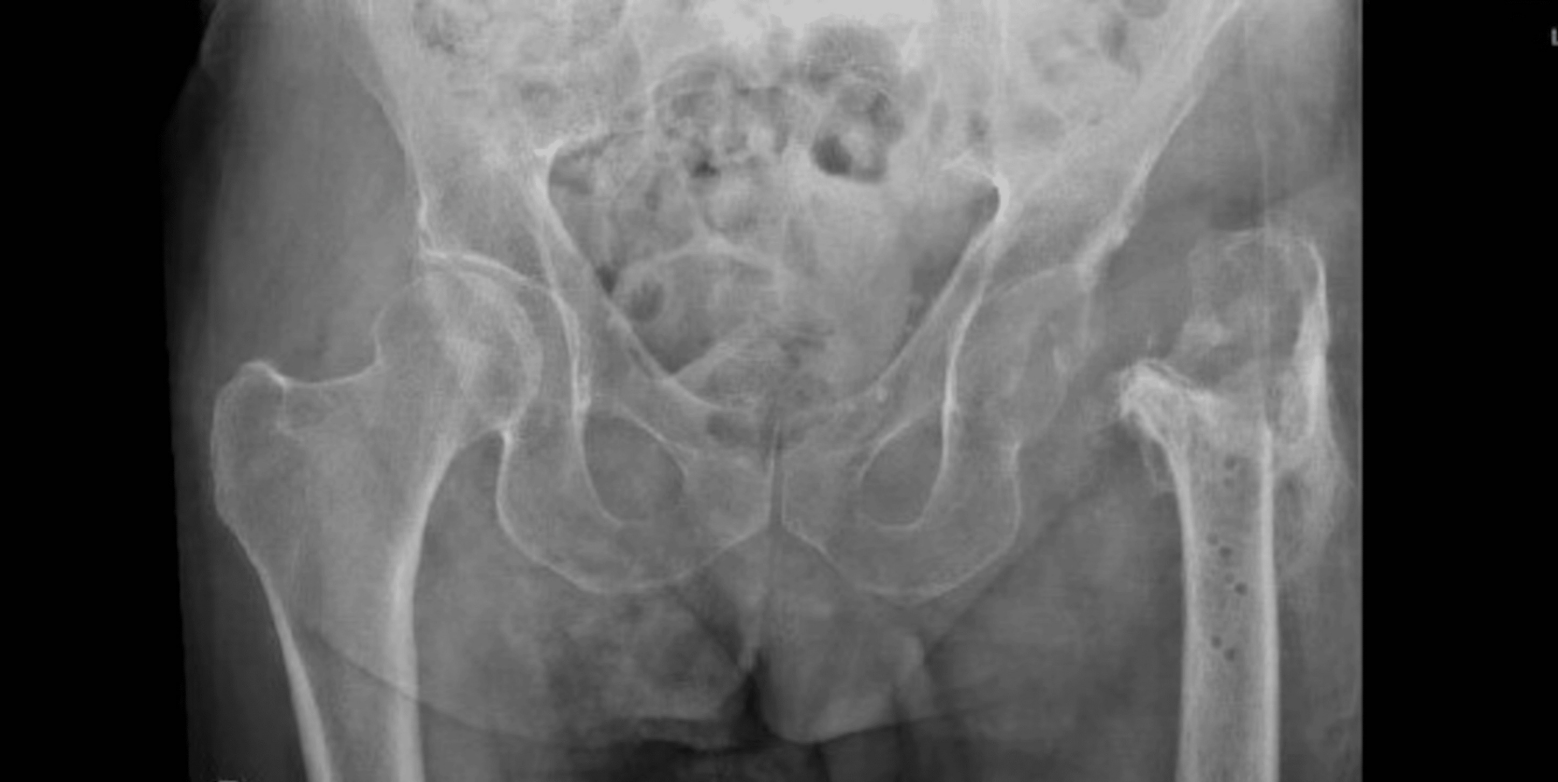
Pelvic X-ray anteroposterior view post-left hip excision arthroplasty

Operative findings included a non-united fracture, depression of the femoral head resting on the posterosuperior acetabulum, significant scar tissue in the acetabulum, a deficient superior acetabular wall, and a deficient posterior femoral neck. Tissue samples and synovial fluid were sent for culture and sensitivity, and the DHS implants were removed with excision of the femoral head (Figure 6).

Intraoperative fluid and tissue samples grew Staphylococcus epidermidis, which was sensitive to vancomycin and resistant to clindamycin and flucloxacillin. The patient was started on intravenous vancomycin. The postoperative course was complicated by wound dehiscence and collection. The patient subsequently underwent two additional wound washouts with debridement of necrotic tissue, during which Stimulan beads impregnated with gentamicin were used for local antimicrobial control.

Intravenous antibiotics were later switched to oral ciprofloxacin and rifampicin after improvement in inflammatory markers, and were prescribed for six weeks. At discharge, the patient was able to transfer from bed to chair with assistance. An outpatient follow-up appointment was arranged; however, the patient was lost to follow-up.

## Discussion

Although very rare, hip dislocation after fracture fixation has been reported to occur spontaneously or secondary to infection or minor trauma. This report highlights a rare but clinically significant complication of a commonly performed procedure, one that many orthopaedic surgeons may never encounter in their careers. In this case, intraoperative images at the time of fixation demonstrated adequate fracture reduction and fixation; the postoperative period was uneventful, and the initial clinical presentation did not suggest infection. Failure of fracture healing could have been due to several factors unrelated to infection. There was no loosening around the implant, and initial image-guided joint aspiration was negative. However, the CRP level was elevated, which could not be explained by other causes. Chest X-ray, urine culture, and blood culture were negative. Intraoperative joint fluid and tissue cultures subsequently grew Staphylococcus epidermidis.

Following antibiotic therapy, the inflammatory markers normalised, and the patient’s clinical condition improved. Tzurbakis et al. and Evans et al. have reported cases of hip dislocation after DHS fixation secondary to deep infection. Tzurbakis et al. described a case with a presentation similar to ours, with an uneventful postoperative course, except that in their case, the fracture had healed [2]. In contrast, Evans et al. reported three cases complicated during the postoperative period [3]. One patient had an early postoperative wound infection, the second had persistent hip pain, and the third developed an infected hematoma.

Tzurbakis et al. reported the case of a 78-year-old patient with posterior hip dislocation who presented six months after trochanteric fracture fixation using a hip screw, with an uneventful postoperative course. The dislocation was attributed to deep infection, and Staphylococcus epidermidis was isolated from the wound samples. The patient was managed with implant removal, excision arthroplasty, and trochanteric skeletal traction to maintain limb length. A cementless total hip replacement was performed after six weeks of antibiotic therapy [2]. Lewis et al., in their study on the earliest signs of postoperative hip infection, reported that radiological signs were more reliable than clinical or laboratory findings, particularly joint space narrowing and acetabular erosion [4].

A few case reports have described atraumatic hip dislocation [5,6,7,8]. The causes of these dislocations have been attributed to anterior capsular detachment [5] or inadequate reduction combined with poor abductor and capsular tissue quality [6], while in other cases, no specific cause was identified. Attempted open reduction and capsular repair was successful in one case [5], whereas in others, re-dislocation required resection arthroplasty [6]. Syed et al. reported anterior hip dislocation following trivial trauma, proposing contributing factors such as valgus malreduction, a comminuted unstable fracture pattern, and capsular stripping. The case was managed with open reduction, removal of the DHS, and uncemented hemiarthroplasty [9]. Shah et al. described a case of progressive anterior dislocation, which was managed with uncemented total hip replacement after excluding infection [10]. Combalia et al., in their case report, presented a comprehensive literature review, encompassing previously reported hip dislocation cases with various fracture patterns and fixation methods [8]. 

This case report is subject to the inherent limitations of retrospective studies, including missing data, and cannot be used to estimate incidence. Various etiologies have been suggested in the literature as causes of hip dislocation following DHS fixation. In the present case, deep infection was confirmed through intraoperative sampling.

## Conclusions

This report demonstrates that latent deep infection can be an unrecognised cause of hip dislocation following DHS fixation, and may be confirmed only through deep tissue sampling. While atraumatic dislocation after DHS fixation has been described, occult infection must be systematically excluded. Thorough intraoperative sampling is essential for diagnosis. Surgical management, including excision arthroplasty or hip replacement, should be individualised based on patient factors, such as age, functional demand, comorbidities, and bone stock.
